# Synthesis, Structure, and Properties of a Copper(II) Binuclear Complex Based on Trifluoromethyl Containing Bis(pyrazolyl)hydrazone

**DOI:** 10.3390/ijms25179414

**Published:** 2024-08-30

**Authors:** Olga G. Shakirova, Tatiana D. Morozova, Yulia S. Kudyakova, Denis N. Bazhin, Natalia V. Kuratieva, Lyubov S. Klyushova, Alexander N. Lavrov, Lyudmila G. Lavrenova

**Affiliations:** 1 Nikolaev Institute of Inorganic Chemistry, Siberian Branch, Russian Academy of Sciences, Novosibirsk 630090, Russia; kuratieva@gmail.com (N.V.K.); lavrov@niic.nsc.ru (A.N.L.); ludm@niic.nsc.ru (L.G.L.); 2 Department of Chemistry and Chemical Technologies, Faculty of Machinery and Chemical Technologies, Federal State Budget Institution of Higher Education Komsomolsk-na-Amure State University, Komsomolsk-on-Amur 681013, Russia; morozova_tatiana_dmitrievna@mail.ru; 3 Postovsky Institute of Organic Synthesis, Ural Branch of the Russian Academy of Sciences, Yekaterinburg 620137, Russia; yu.kudyakova@gmail.com (Y.S.K.); dnbazhin@gmail.com (D.N.B.); 4 Department of Organic and Biomolecular Chemistry, Institute of Chemical Technology, Ural Federal University Named after the First President of Russia B.N. Yeltsin, Mira Str. 19, Yekaterinburg 620002, Russia; 5 Research Institute of Molecular Biology and Biophysics, FRC FTM, 2/12, Timakova Str., Novosibirsk 630060, Russia; klyushovals@mail.ru

**Keywords:** dinuclear copper(II) complex, (trifluoromethyl)pyrazole, ketazine, single-crystal X-ray diffraction analysis, IR spectroscopy, magnetic susceptibility, cytotoxic properties

## Abstract

A new complex of copper(II) with methyl-5-(trifluoromethyl)pyrazol-3-yl-ketazine (H_2_L) was synthesized with the composition [Cu_2_L_2_]∙C_2_H_5_OH (**1**). Recrystallization of the sample from DMSO yielded a single crystal of the composition [Cu_2_L_2_((CH_3_)_2_SO)] (**2**). The coordination compounds were studied by single-crystal X-ray diffraction analysis, IR spectroscopy, and static magnetic susceptibility method. The data obtained indicate that the polydentate ligand is coordinated by both acyclic nitrogen and heterocyclic nitrogen atoms. The cytotoxic activity of the ligand and complex **1** was investigated on human cell lines MCF7 (breast adenocarcinoma), Hep2 (laryngeal carcinoma), A549 (lung carcinoma), HepG2 (hepatocellular carcinoma), and MRC5 (non-tumor lung fibroblasts). The complex was shown to have a pronounced dose-dependent cytotoxicity towards these cell lines with LC_50_ values in the range of 0.18–4.03 μM.

## 1. Introduction

Drug discovery based on pyrazole scaffold is one of the popular trends in medicinal chemistry [1,2,3,4,5]. Substituted pyrazole derivatives demonstrate a broad spectrum of biological properties, including antibacterial, cytotoxic, anti-inflammatory, antitubercular, antitumor activities, etc. [6,7,8,9,10,11,12,13,14,15,16,17]. In some cases, pyrazole-bearing pharmacological agents display multiple actions against different targets.

**Figure 1:**
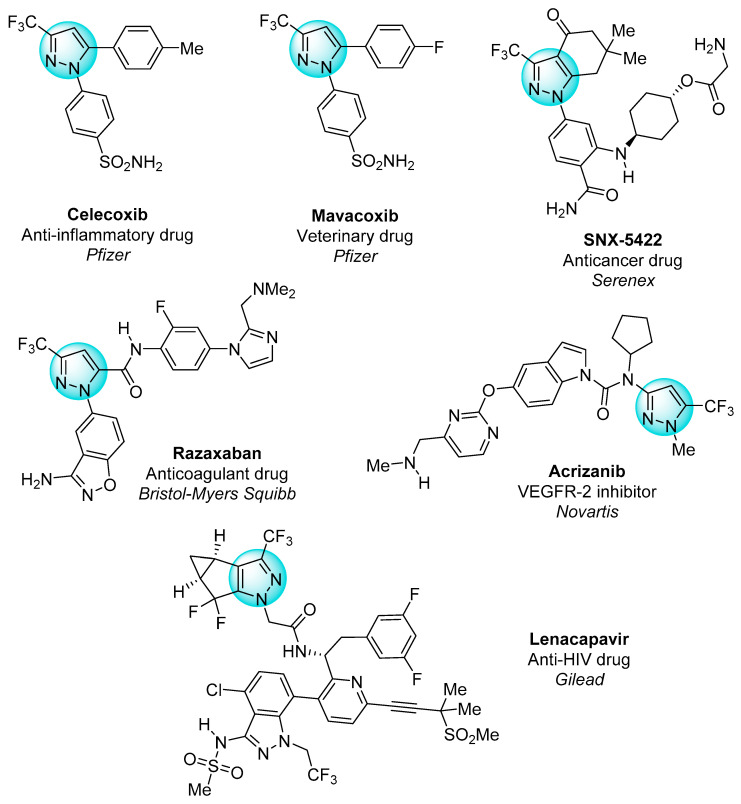
Pharmaceuticals with the trifluoromethylated pyrazole motif.

The functionalization of the pyrazole core with fluorinated substituents has led to the development of many bioactive molecules, as shown in Figure 1 [13,15,16,17,18]. Currently, almost a quarter of all drugs in the pharmacological market contain fluorine in their composition. Owing to the high electronegativity, small size, and low polarizability of fluorine, its introduction into the structure of organic compounds leads to an increase in their lipophilicity, membrane permeability, and metabolic stability, which determines pronounced biological activity, including anticancer [19].

The use of pyrazoles in the synthesis of metal complexes represents a promising avenue in the design of novel antitumor agents [20]. This strategy enables the combination of pyrazole bioactivity with their coordination properties as N-ligands [21,22,23,24]. Complexes based on the biocompatible copper(II) ion represent an alternative to existing anticancer metallodrugs [20]. Depending on the nature of substituents and reaction conditions, pyrazoles behave both as mono- and bidentate ligands, forming complexes with copper ions of various oxidation states and nuclearity [20,21,22,23,24,25,26,27,28,29]. In particular, the presence of trifluoromethyl groups in the pyrazole ring leads to an increase in the N–H acidity of the heterocycle and the stability of complexes with transition metals [13,30]. In addition, copper(II) pyrazolates are of interest because of their magnetic, catalytic, and photophysical properties [31,32,33,34,35].

**Figure 2:**
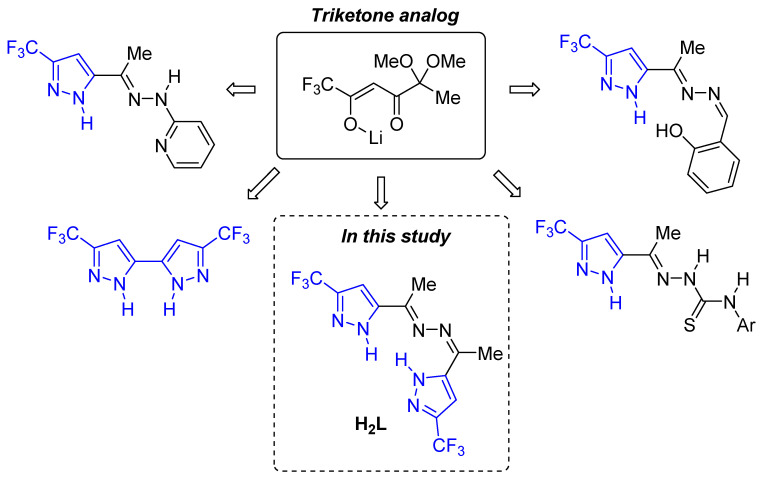
Functionalized pyrazoles and bis-pyrazoles based on analogs of 1,2,4-triketones.

Previously, we have developed an approach to the synthesis of functionalized pyrazoles based on analogs of 1,2,4-triketones [18,33,34,35,36,37,38,39,40] (Figure 2), including fluorine-containing bis-pyrazoles [39,40]. In this work, we have investigated the complexation reactions of copper(II) with methyl-5-(trifluoromethyl)pyrazol-3-yl-ketazine (**H_2_L**) (Figure 2). The structure of **H_2_L** contains six nitrogen-donor atoms capable of coordination with transition metal ions. Although the potential of pyrazole derivatives in coordination chemistry is well documented, the number of fluorinated bis-pyrazoles used as ligands remains low [13].

## 2. Results and Discussion

### 2.1. General Procedure for Synthesis of Compounds

**Scheme 1:**
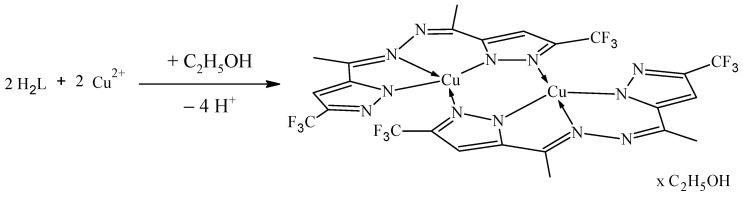
Synthesis of complex **1**.

Complex **1** was synthesized by varying the metal: ligand ratios (2:1, 1:1, 1:2, 1:3) and using solutions of different copper(II) salts—nitrate, chloride, and sulfate. In all cases, the same dark green color complex was isolated. Elemental analysis and IR spectra of the complexes obtained with different copper(II) salts showed that they have the same composition [Cu_2_L_2_]∙C_2_H_5_OH (Scheme 1).

**Scheme 2:**
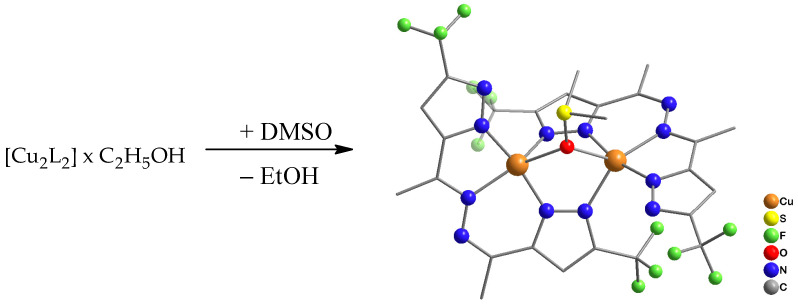
Synthesis of complex **2**.

Upon recrystallization of portion **1** from DMSO and prolonged standing of the solution, we were able to obtain a single crystal of the complex [Cu_2_L_2_((CH_3_)_2_SO)] (**2**) suitable for X-ray diffraction analysis (Scheme 2).

### 2.2. Crystallography

X-ray phase analysis data indicate the crystallinity of powdered sample **1**. However, we were unable to grow single crystals of **1** suitable for analysis. To determine the coordination ability of the new ligand, we obtained and studied a single crystal of complex **2**. It should be noted that the comparison of diffractograms indicates that (despite the same Cu:ligand ratio) complexes **1** and **2** are not isostructural (Figure S1). The differences should be explained, among other things, by their different composition and packaging of molecules.

**Figure 3:**
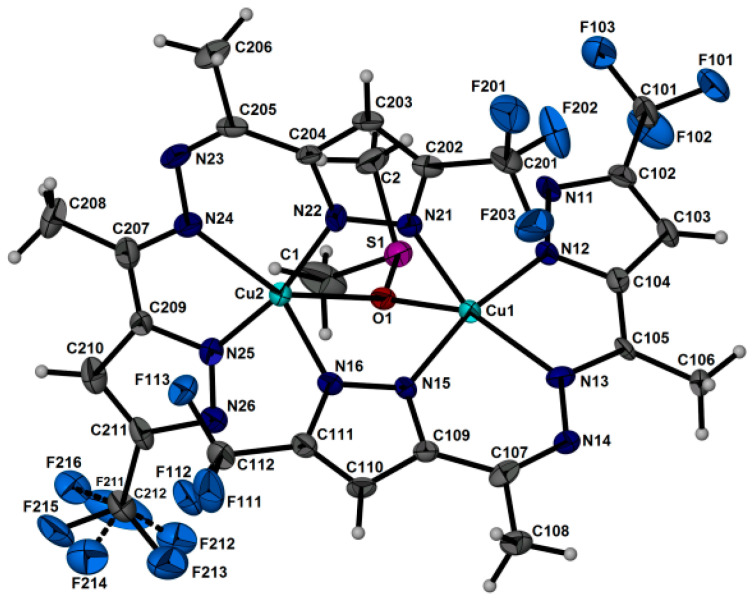
The molecular complex **2** with the labeling (ellipsoids are plotted with 50% probability).

**Figure 4:**
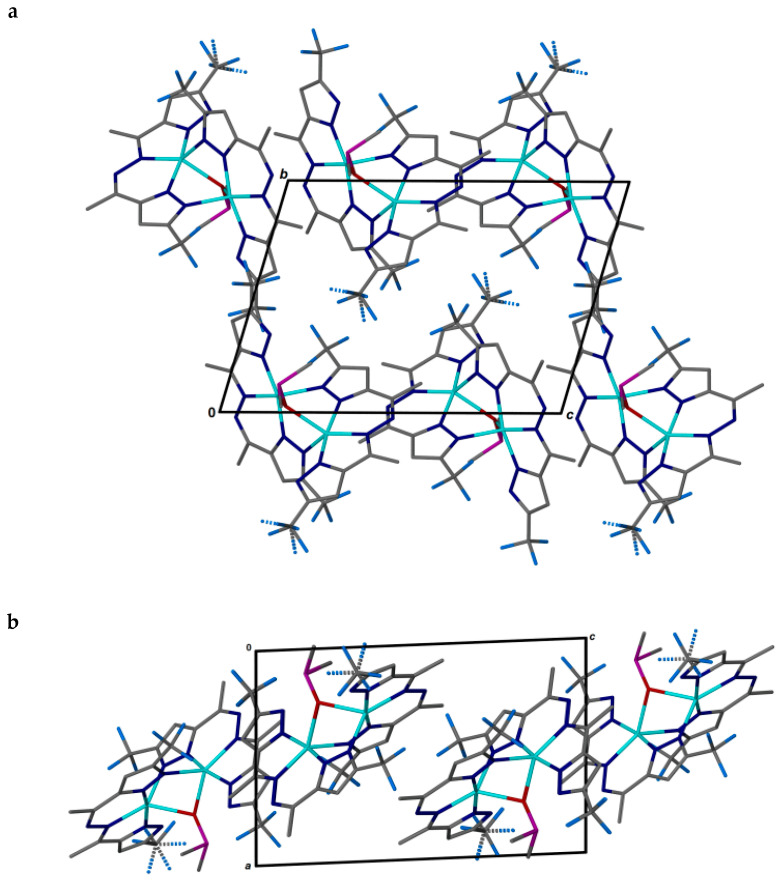
Two orientations of the pseudo-layer of **2** in the *bc* plane (**a**) and along the b-axis (**b**).

Single-crystal X-ray diffraction analysis of complex **2** indicates that the [Cu_2_L_2_((CH_3_)_2_SO)] phase crystallizes in a triclinic crystal system. The crystal packing is molecular (Figure 3 and Figure 4). Copper(II) ions replace both protons in the ligand **H_2_L**, and the obtained anion L^2−^ acts as a tetradentate ligand, coordinating to two Cu(II) ions by nitrogen atoms; at the same time, the carbon-nitrogen framework of the ligand remains almost planar. The coordination sphere of each Cu(II) can be described as a slightly inclined trigonal bipyramidal geometry with 4N atoms from two ligands and an O atom from DMSO. The equatorial planes of the bipyramids defined by Cu(1), O(1), N(13), N(21) [N(13)-Cu(1)-N(21) = 135.5(2), N(13)-Cu(1)-O(1) = 139.5(2), N(21)-Cu(1)-O(1) = 84.9(2); totally 359.9(6)°] and Cu(2), O(1), N(16), N(24) [N(24)-Cu(2)-N(16) = 141.4(2), N(16)-Cu(2)-O(1) = 84.94(18), N(24)-Cu(2)-O(1) = 133.61(19); totally 359.9(7)°] are absolutely planar. A five-membered CuN_2_C_2_ chelate cycle is formed using one nitrogen atom of the first pyrazole ring and one nitrogen of the diazo group. Two nitrogen atoms of the second pyrazole ring form the six-membered metallocycle Cu_2_N_4_, acting as a bridge. The O atom of the solvent DMSO molecule coordinates with both Cu(II) ions, serving as an additional stability bridge. The main Cu-N(O) interatomic distances and valence angles are presented in Table 1.

The corrugated pseudo-layers of molecular binuclear complex particles in the bc plane, distorted according to the elongated shape, could be isolated in the package (Figure 4). Further packing of the layers takes place one above the other without displacement, according to the corrugation.

**Scheme 3:**
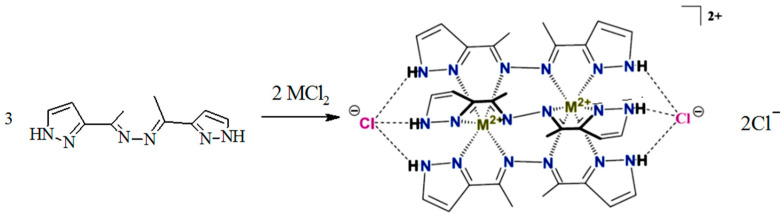
The expected type of cage metal complexes for 3D metals with non-fluorinated ligands [41].

It should be noted that the coordination ability of the fluorinated ligand differs significantly from that assumed for the non-fluorinated analog [41] (Scheme 3).

### 2.3. Spectroscopy

In the high-frequency region of the infrared spectra of the ligand (Figure S2), (N-H) vibration bands near 3250 cm^−1^ are observed. In the spectrum of complex **1** (Figure S3), (O-H) bands are present within the broad range of 3500–3000 cm^−1^. The (C-H) bands of methyl groups and pyrazole rings are in the interval 3090–2830 cm^−1^. For **H_2_L**, at 1630 cm^−1^, a band of valence-deformation vibration of the acyclic bond -C=N- is observed, which is very sensitive to coordination. In the spectrum of the complex, this band appears at 1608 cm^−1^. In the spectrum of **H_2_L** in the region of 1550–1500 cm^−1^, the bands of valence-deformation vibrations of pyrazole rings are observed, which are split or shifted by ~30 cm^−1^ in the spectrum of the complex (Table 2). The changes in the position and shape of the bands are caused by the coordination of nitrogen atoms to Cu(II) [42].

### 2.4. Magnetic Measurements

**Figure 5:**
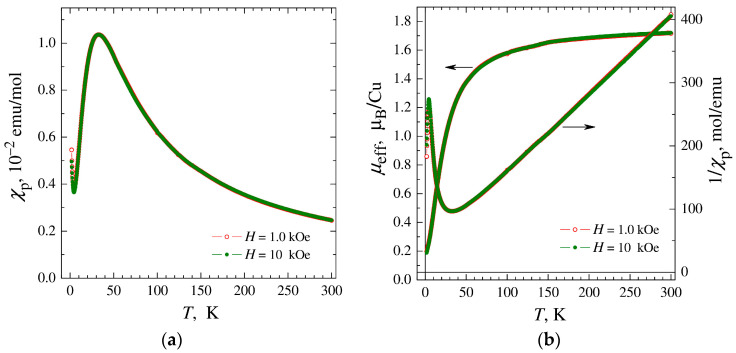
(**a**) Temperature dependences of paramagnetic component of magnetic susceptibility χ_p_ of complex **1** measured in magnetic fields H = 1, 10 kOe. (**b**) Temperature dependences of the inverse susceptibility 1/χ_p_ and effective magnetic moment μ_eff_ calculated in the approximation of non-interacting ions (θ = 0).

The temperature and field dependences of the magnetic susceptibility were measured for a polycrystalline sample of complex **1** and corrected for temperature-independent Langevin diamagnetism to single out the paramagnetic component of the susceptibility χ_p_(T) associated with copper ions. The resulting χ_p_(T) dependences were insensitive to the thermo-magnetic prehistory and exhibited a broad peak at T_m_ ≅ 34 K, independent of the magnitude of the applied magnetic field (Figure 5a). The drop in magnetic susceptibility upon cooling below Tm is unambiguous evidence of the predominance of antiferromagnetic (AF) interactions between copper ions, while the absence of phase transition and the smoothness of χ_p_ change indicate a reduced dimensionality of the magnetic subsystem, reduced to the level of chains or polynuclear clusters.

In the high-temperature region, the obtained χ_p_(T) data can be formally approximated by the Curie–Weiss dependence $\chi_{p}(T)=N_{A}\mu_{\mathrm{eff}}^{2}/3k_{B}(T-\theta )$ with values of the effective momentum μ_eff_ ≈ 1.80 μ_B_ and the Weiss constant θ ≈ −26 K (Figure 5b). The μ_eff_ value is characteristic of copper ions Cu^2+^ (S = 1/2) and corresponds to an average value of the g-factor g ≈ 2.08, exceeding the spin-only value g ≈ 2 due to the contribution of orbital moments. In turn, in the region of the lowest temperatures, after passing the peak, a susceptibility increase is observed, typical for most samples of chain and polynuclear complexes and associated with the inevitable presence of a small number of mononuclear impurities. To determine the percentage of monomer impurities, their contribution to the temperature dependence of the susceptibility was approximated by the Curie–Weiss dependence and the contribution to the field dependence of the magnetization M(H) was approximated by the Brillouin function. The analysis showed that the monomer fraction includes ~1.2% of copper ions. The magnetic susceptibility of the main phase can be determined by subtracting the contribution of mononuclear impurities from the experimental data.

**Figure 6:**
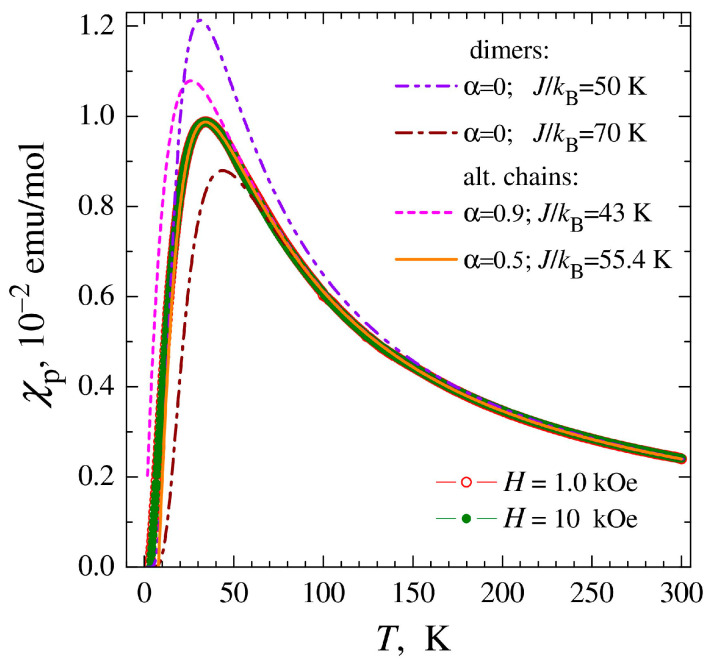
Temperature dependencies of the magnetic susceptibility χ_p_ of complex 1, after subtracting the contribution from the monomer impurity. The lines show approximations of the experimental data by theoretical models: the model of non-interacting dimers (dashed-dotted line, dashed-double dotted line) and the model of dimerized chains (dashed and solid lines) with the interaction parameters within dimers *J* and between dimers *J*′ = α*J* indicated in the figure. The first curve (dashed-double dotted line) corresponds to the optimization of the model description in the region of the magnetic susceptibility peak; for the other curves, the fitting of the J parameter was carried out in the temperature region 80–300 K.

As can be seen in Figure 6, the magnetic susceptibility of the main phase of complex **1** after passing the peak at T_m_ decreases almost to zero, which clearly indicates the formation of a gap in the spectrum of spin excitations. The most obvious explanation for this is related to the presence of magnetic dimers in the structure of the complex described by the Hamiltonian $H=-J{\vec{S}}_{1}\cdot {\vec{S}}_{2}$ [43], where J is the exchange interaction between Cu^2+^ ions inside the dimer (J < 0 for the AF interaction), which is consistent with the structural data for the related complex **2**. However, the model of isolated dimers, in which the interaction between dimers J′ = αJ is assumed to be zero, does not allow us to achieve any acceptable quality description of the experimental data χ_p_(T) (Figure 6). The position of the magnetic susceptibility peak at T_m_ ~ 34 K in the isolated dimer model corresponds to the exchange interaction within the dimers J/k_B_ ≈ 50 K; however, when such a value of J is chosen, the model calculation significantly exceeds the experimental values of χ_p_ (dashed-double dotted curve in Figure 6). In turn, the behavior of the magnetic susceptibility within the range of 80–300 K in the same model requires a value of J/k_B_ ≈ 70 K (dashed-dotted line). This can be explained by the presence of an additional AF interaction between the dimers, J′ = αJ ≠ 0.

One of the limiting cases of strong AF interaction between dimers is the homogeneous chain, which also exhibits a broad peak in magnetic susceptibility [44]. However, the homogeneous chain lacks a gap in the magnetic excitation spectrum, and the magnetic susceptibility drops at low temperatures by only ~30% relative to the peak value rather than to zero, as observed experimentally. An intermediate variant is a partially dimerized chain described by the Hamiltonian $H=-J\sum_{i}^{n/2}\left({\vec{S}}_{2i-1}\cdot {\vec{S}}_{2i}+\alpha {\vec{S}}_{2i}\cdot {\vec{S}}_{2i+1}\right)$ [44,45,46], when a strong AF interaction in pairs of ions, J, alternates with a weaker J′ = αJ interaction between pairs. The availability of the theory of partially dimerized chains [44,45] allowed us to perform simulations that showed that by adjusting the degree of dimerization α (dashed and solid lines in Figure 6), a reasonably good agreement of the model with the experimental data can be achieved. The optimal fitting result was obtained for α ≈ 0.5, which means a sufficiently strong interaction between dimers, only two times weaker than the AF interaction within the dimer, J′ = J/2.

Detailed structural data (Figure 3) were obtained only for complex **2** since it was possible to grow single crystals for it. However, certain conclusions can be drawn from the structure of complex **1**, which differs from complex **2** only by the absence of the (CH_3_)_2_SO group coordinated with the copper(II) ion. As can be seen in Figure 3, the two copper ions in the structure of complex **2**, and obviously complex **1**, are united in a stable metallocycle Cu_2_N_4_, which leads to the appearance of magnetic dimers. In the structure of complex **2**, dimers are spatially separated by groups (CH_3_)_2_SO, while in complex **1**, these groups are absent, which leads, according to the magnetic data, to the formation of a chemical bond between the dimers, capable of transferring between them strong enough AF exchange interaction. It should be noted that the magnetic susceptibility data, while demonstrating the presence of interaction between magnetic Cu^2+^-Cu^2+^ dimers, still do not allow us to conclude whether linear chains of dimers, ladder, or other similar structures are formed upon the packing of molecules in the crystal structure.

### 2.5. Cytotoxic Activity

**Figure 7:**
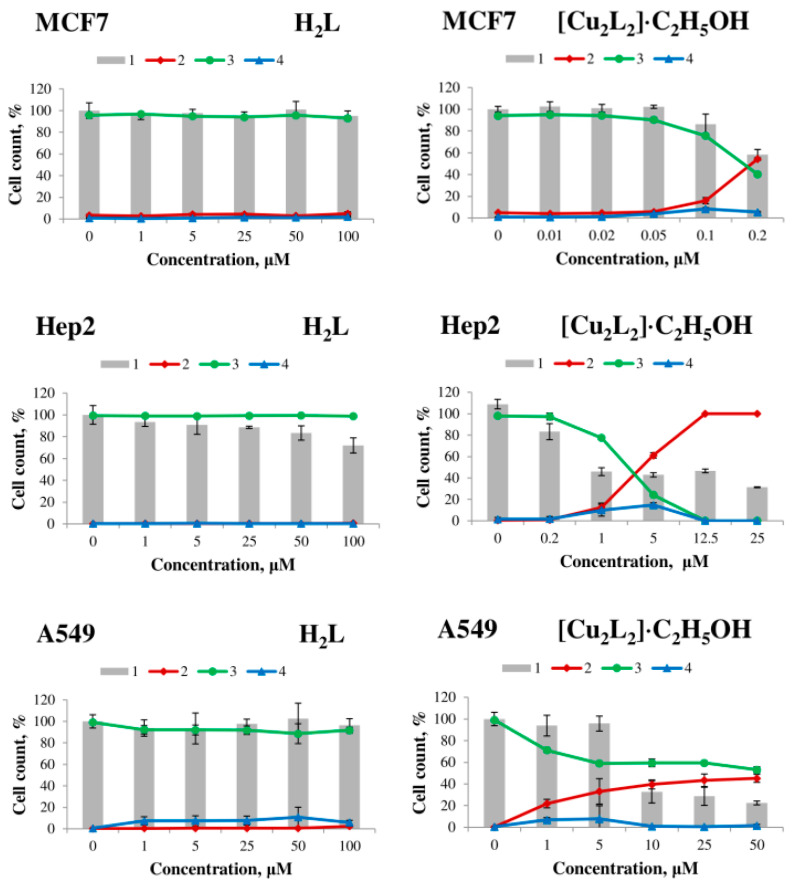

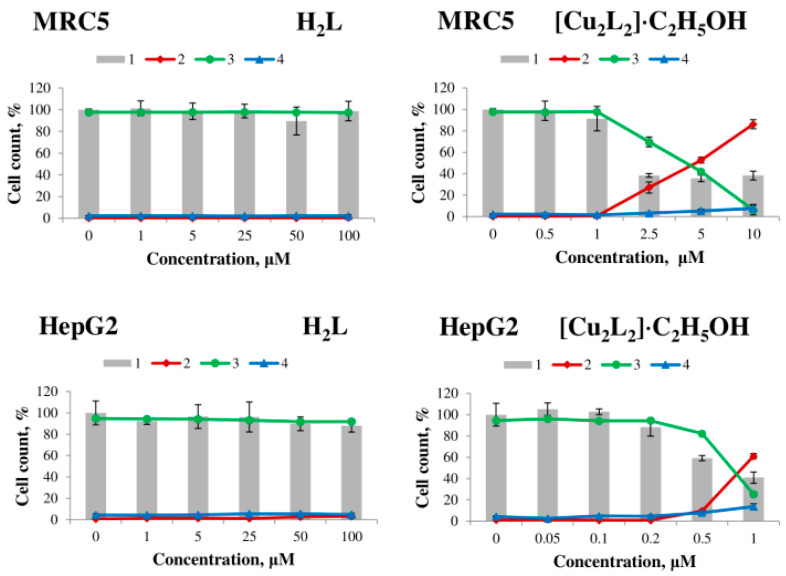
Effect of **H_2_L** ligand (**left**) and [Cu_2_L_2_]∙C_2_H_5_OH complex (**right**) on the viability of MCF7, Hep2, A549 human tumor cells, MRC5 human non-tumor fibroblasts and HepG2 after 48 h exposure. 1—total number of cells, 2—dead cells, 3—live cells, 4—apoptotic cells.

The effect of **H_2_L** ligand, Cu(NO_3_)_2,_ and complex **1** on human cell viability was evaluated on the tumor cell lines MCF7 (breast adenocarcinoma), Hep2 (laryngeal carcinoma), A549 (lung carcinoma), HepG2 (hepatocellular carcinoma) and non-tumor MRC5 lung fibroblasts. The results of the study are presented in Table 3 in the form of the LC50 parameter, which is calculated as the concentration of the substance at which cell death is 50%, and Figure 7 and Figure S4.

The **H_2_L** ligand and copper (II) salt have no cytotoxic effect on all cell lines after 48 h of exposure in the concentration range from 1 to 100 μM, whereas the [Cu_2_L_2_]∙C_2_H_5_OH complex exhibits strong dose-dependent cytotoxic activity (Table 3, Figure 7). Complex **1** exhibited the highest activity on MCF7 cells (LC_50_ = 0.18 ± 0.03 μM) and the lowest activity on A549 cells (LC_50_ = 43.6 ± 2.6 μM).

Based on the obtained data on cytotoxic activity, the selectivity indices (**SI**, it is a ratio of the LC_50_ value for non-tumor MRC5 fibroblasts to the LC_50_ value for tumor cells) of the action of complex **1** against tumor cells were calculated, and the results are presented in Table 3. According to the literature, compounds with **SI** > 3 (or >9 in some sources) are considered promising for further studies as potential antitumor agents [47]. The investigated copper(II) complex shows high selectivity of action against HepG2 (**SI** = 5.1) and MCF7 (**SI** = 22.4) cells; for Hep2 cells, the activity of the complex is comparable to the activity on non-tumor fibroblasts MRC5, and for A549 cells **SI** < 1.

## 3. Materials and Methods

### 3.1. Synthetic Procedures

Synthesis of [Cu_2_L_2_]∙C_2_H_5_OH (**1**). A 0.176 g (0.50 mmol) portion of **H_2_L** was dissolved on heating in 15 mL of ethanol, and 0.060 g (0.25 mmol) of Cu(NO_3_)_2_∙3H_2_O was dissolved in 1 mL of H_2_O with the addition of 1 drop of 1N HNO_3_. The copper(II) solution was added to the hot ligand solution, and a dark green solution was formed, from which a precipitate of the same color quickly formed. The precipitate was filtered off the next day, washed several times with ethanol, and air-dried. The yield was 0.08 g (73% based on Cu).

For **1** found (%): C 35.3, H 2.6, N 18.7, Cu = 14.5.

For C_26_H_22_Cu_2_F_12_N_12_O, calculated (%): C 35.7, H 2.8, N 19.2, Cu 14.6.

### 3.2. Materials and Techniques

Commercially available reagents and solvents were used for the synthesis without additional purification. Methyl-5-(trifluoromethyl)pyrazol-3-yl-ketazine (**H_2_L**) was prepared according to the published method [40].

Elemental analysis for C, H, N was performed in the Laboratory of Microanalysis of the N.N. Vorozhtsov Novosibirsk Institute of Organic Chemistry SB RAS. Copper(II) content was analyzed complexometrically with EDTA disodium salt solution and murexide as an indicator after decomposition of the complex samples in concentrated acids (H_2_SO_4_ + HClO_4_).

### 3.3. Crystallography

X-ray powder diffraction data were collected on a Shimadzu XRD 7000 diffractometer (CuK_α_ radiation (λ = 1.5406 Å), Ni filter, scintillation detector) at room temperature.

Single-crystal X-ray diffraction data were collected using the graphite monochromatized MoKα-radiation (λ = 0.71073 Å) at 150(2) K on an X8APEX Bruker Nonius diffractometer equipped with a 4K CCD area detector. The φ-scan technique was employed to measure intensities. Absorption corrections were applied empirically using the SADABS program [48]. Structures were solved by the direct methods of the difference Fourier synthesis and further refined by the full-matrix least squares method using the SHELXTL package(Veesion 2014/7) [49]. Atomic thermal parameters for non-hydrogen atoms were refined anisotropically (Table 4). The positions of hydrogen atoms were calculated corresponding to their geometrical conditions and refined using the riding model. The disordering of the CF_3_ group was introduced statistically with the restriction of C-F bonds with a length of 1.32(1) Å and a population of 80.8/19.2%. The introduction of the second position of the fluorine atoms also required a slight limitation of the thermal displacement parameters (ISOR).

### 3.4. Spectroscopy

IR absorption spectra were taken on an IRAfinity-1S spectrometer (Shimadzu, Kyoto, Japan) 4000–400 cm^−1^ at room temperature, and samples were prepared in KBr.

### 3.5. Magnetic Measurements

Magnetic properties were measured on a Quantum Design MPMS-XL SQUID magnetometer within the temperature range of 1.77–300 K at magnetic fields H = 0–10 kOe. To determine the paramagnetic component of the molar magnetic susceptibility χ_p_(T), the contributions of core diamagnetism χ_d_ and possible ferromagnetism of micro-impurities χ_FM_ were subtracted from the measured values of the total susceptibility χ = M/H (M = magnetization). The temperature-independent contribution χ_d_ was calculated according to Pascal’s additive scheme. To check the presence of ferromagnetic contribution χ_FM_, the field dependences M(H) and temperature dependences M(T) at different values of the magnetic field were measured, after which the total magnetization of the sample was decomposed into ferromagnetic and paramagnetic components. For the studied sample, the ferromagnetic contribution to the magnetization at H = 10 kOe did not exceed 2%.

### 3.6. Cytotoxic Activity

The human lung carcinoma cell line A549 was purchased from Biolot (Russia), non-tumor human lung fibroblasts MRC5, breast adenocarcinoma MCF7, laryngeal carcinoma Hep2, hepatocellular carcinoma HepG2—from State Scientific Center of Virology and Biotechnology “Vector”. Cell viability was assessed by double staining Hoechst 33342/propidium iodide (PI) [50]. Cells were seeded onto 96-well plates and cultured in DMEM (MRC5, MCF7, Hep2, HepG2) or DMEM/F12 (A549) medium in a CO_2_ incubator at 37 °C. After 24 h, drugs dissolved in ethanol were added at a concentration range of 0.01–100 μM and incubated for 48 h. Cells were stained with fluorescent dyes Hoechst 33342 (Sigma-Aldrich) and propidium iodide (Invitrogen) for 30 min at 37 °C. Imaging was performed on an IN CellAnalyzer 2200 instrument (GE Healthcare UK Limited Amersham Place Little Chalfont Buckinghamshire HP7 9NA UK) in automatic mode at 4 fields per well. The obtained images were analyzed using “InCellInvestigator” software(Version 1.5). The result is presented as the percentage of live, dead, and apoptotic cells in the whole population from three independent experiments ± standard deviation. The experimental dependence of the percentage of live cells on the concentration of the compound was approximated by a nonlinear function; the LC50 parameter was calculated as the concentration of the compound at which cell death is 50%.

## 4. Conclusions

Thus, this pioneering work showed that N-donor ligands based on bis(pyrazolyl)hydrazone containing fluorinated substituents are promising for the synthesis of Cu(II) molecular complexes with significant magnetic and biological activities. It has been shown that the system is not sensitive to the stoichiometry of synthesis and counterions but is sensitive to solvent.

A new copper(II) complex with ligand L^2-^, which is formed by the loss of two protons from methyl-5-(trifluoromethyl)pyrazol-3-yl-ketazine, was synthesized with the composition [Cu_2_L_2_]∙C_2_H_5_OH. Recrystallization of the isolated complex from DMSO yielded a single crystal of the complex of the composition [Cu_2_L_2_((CH_3_)_2_SO)]. Single-crystal X-ray diffraction data showed that the complex [Cu_2_L_2_((CH_3_)_2_SO)] has a binuclear structure. Ligand L^2-^ is polydentate and is coordinated by both heterocyclic and acyclic nitrogen atoms.

Magnetochemical studies of the [Cu_2_L_2_]∙C_2_H_5_OH complex have shown that copper(II) ions arranged in Cu_2_N_4_ metallocycles form Cu^2+^-Cu^2+^ AF dimers with a relatively strong, J/k_B_ ≈ 50 K exchange interaction. When the molecules of the complex are packed into a crystal structure, chemical bonds are formed between them, which are also capable of transferring the AF exchange interaction between the dimers.

The [Cu_2_L_2_]∙C_2_H_5_OH complex has a strong cytotoxic effect on MCF7, Hep2, HepG2, and MRC5 human cells and exhibits high selectivity for HepG2 (**SI** = 5.1) and MCF7 (**SI** = 22.4) tumor cell lines. The nature of this impact requires further research, which will be continued.

## Figures and Tables

**Table 1:** Basic interatomic distances (A) and angles (°) for [Cu_2_L_2_((CH_3_)_2_SO)] (**2**).

Bond	d, Å	Angle	°	Angle	°
Cu(1)-N(15)	1.912(4)	N(15)-Cu(1)-N(12)	167.1(2)	N(22)-Cu(2)-N(25)	167.1(2)
Cu(1)-N(12)	1.929(5)	N(15)-Cu(1)-N(13)	88.8(2)	N(22)-Cu(2)-N(24)	87.9(2)
Cu(1)-N(13)	2.044(6)	N(12)-Cu(1)-N(13)	81.3(2)	N(25)-Cu(2)-N(24)	80.7(2)
Cu(1)-N(21)	2.097(6)	N(15)-Cu(1)-N(21)	92.4(2)	N(22)-Cu(2)-N(16)	94.7(2)
Cu(1)-O(1)	2.260(4)	N(12)-Cu(1)-N(21)	100.4(2)	N(25)-Cu(2)-N(16)	98.0(2)
Cu(2)-N(22)	1.937(5)	N(13)-Cu(1)-N(21)	135.5(2)	N(24)-Cu(2)-N(16)	141.4(2)
Cu(2)-N(25)	1.944(6)	N(15)-Cu(1)-O(1)	86.35(19)	N(22)-Cu(2)-O(1)	84.84(18)
Cu(2)-N(24)	2.052(6)	N(12)-Cu(1)-O(1)	95.80(19)	N(25)-Cu(2)-O(1)	98.7(2)
Cu(2)-N(16)	2.062(6)	N(13)-Cu(1)-O(1)	139.5(2)	N(24)-Cu(2)-O(1)	133.61(19)
Cu(2)-O(1)	2.274(4)	N(21)-Cu(1)-O(1)	84.9(2)	N(16)-Cu(2)-O(1)	84.94(18)

**Table 2:** Wave numbers (frequencies, cm^–1^) of absorption band maxima in the IR spectra of **H_2_L** and complex **1**.

Assignment	H_2_L	1
ν (О–Н)		3367, 3229
ν (N–Н)	3252	
ν (Cring–H)	3160, 3057	3136
ν (CH_3_)	2986, 2930	2974, 2930
ν (–C=N–)	1630	1608
R(pz)	1559	1560, 1525
ν (С–H) (scissoring)	1496, 1445, 1413, 1374, 1312	1497, 1453, 1434, 1375, 1352
ν (CF_3_)	1258, 1203, 1175, 1120	1253, 1156, 1124, 1095
ν (С–H) (twisting)	1077, 1006, 977, 819	1055, 989, 811
ν (С–H) (rocking)	748, 717, 681	772, 760, 656, 623

**Table 3:** Cytotoxic activity of the ligand and complex **1** expressed in terms of LC_50_ parameter and selectivity index (**SI**).

Compound	LC_50_, μМ					SI			
	HepG2	Hep2	MCF7	A549	MRC5	HepG2	Hep2	MCF7	A549
**Cu(NO_3_)_2_**	>100	>100	>100	-	>100	-	-	-	-
**Н_2_L**	>100	>100	>100	>100	>100	-	-	-	-
**1**	0.79 ± 0.01	2.53 ± 0.03	0.18 ± 0.03	43.6 ± 2.6	4.03 ± 0.13	5.1	1.6	22.4	0.1

**Table 4:** Crystallographic data and conditions of the diffraction experiment for [Cu_2_L_2_((CH_3_)_2_SO)] (**2**).

CCDC		2361180
Empirical formula		C_26_H_22_Cu_2_F_12_N_12_OS
Formula weight		905.69
Crystal system		triclinic
Space group		*P*–1
Cell Parameters	*a*, Å	10.0089(17)
	*b*, Å	11.3575(13)
	*c*, Å	15.616(3)
	*A*, °	73.830(5)
	*β*, °	88.420(3)
	*γ*, °	76.405(3)
V, Å^3^		1655.8(4)
Z		2
Calc. density, g/cm^3^		1.817
μ, mm^−1^		1.460
Crystal size, mm^3^		0.320 × 0.100 × 0.030
Scan range, *θ*, degree		2.451–26.022
Range h, k, l		−12 ≤ h ≤ 12, −10 ≤ k ≤ 14, −19 ≤ l ≤ 19
Reflections collected/independent (R_int_)		11,603/6339 (0.0690)
Completeness, %		97.3
Data/restraints/parameters		6339/23/522
Goodness-of-fit		1.034
R_1_, wR_2_ (I > 2σ(I))		0.0716, 0.1458
R_1_, wR_2_ (all data)		0.1327, 0.1654
Δρ_max_, Δρ_min_, eÅ^–3^		0.583, −0.637

## Data Availability

The data presented in this study are available on request from the corresponding author.

## Supplementary Materials

The following supporting information can be downloaded at: https://www.mdpi.com/article/10.3390/ijms25179414/s1.
