# Supplementary material for: Synthesis, Structure, and Properties of a Copper(II) Binuclear Complex Based on Trifluoromethyl Containing Bis(pyrazolyl)hydrazone
Source: Int J Mol Sci. 2024 Aug 30;25(17):9414. doi: 10.3390/ijms25179414 (PMC11395124; doi:10.3390/ijms25179414)
Supplement: Supplementary file 1 [file ijms-25-09414-s001.zip › ijms-3160629-supplementary.pdf]

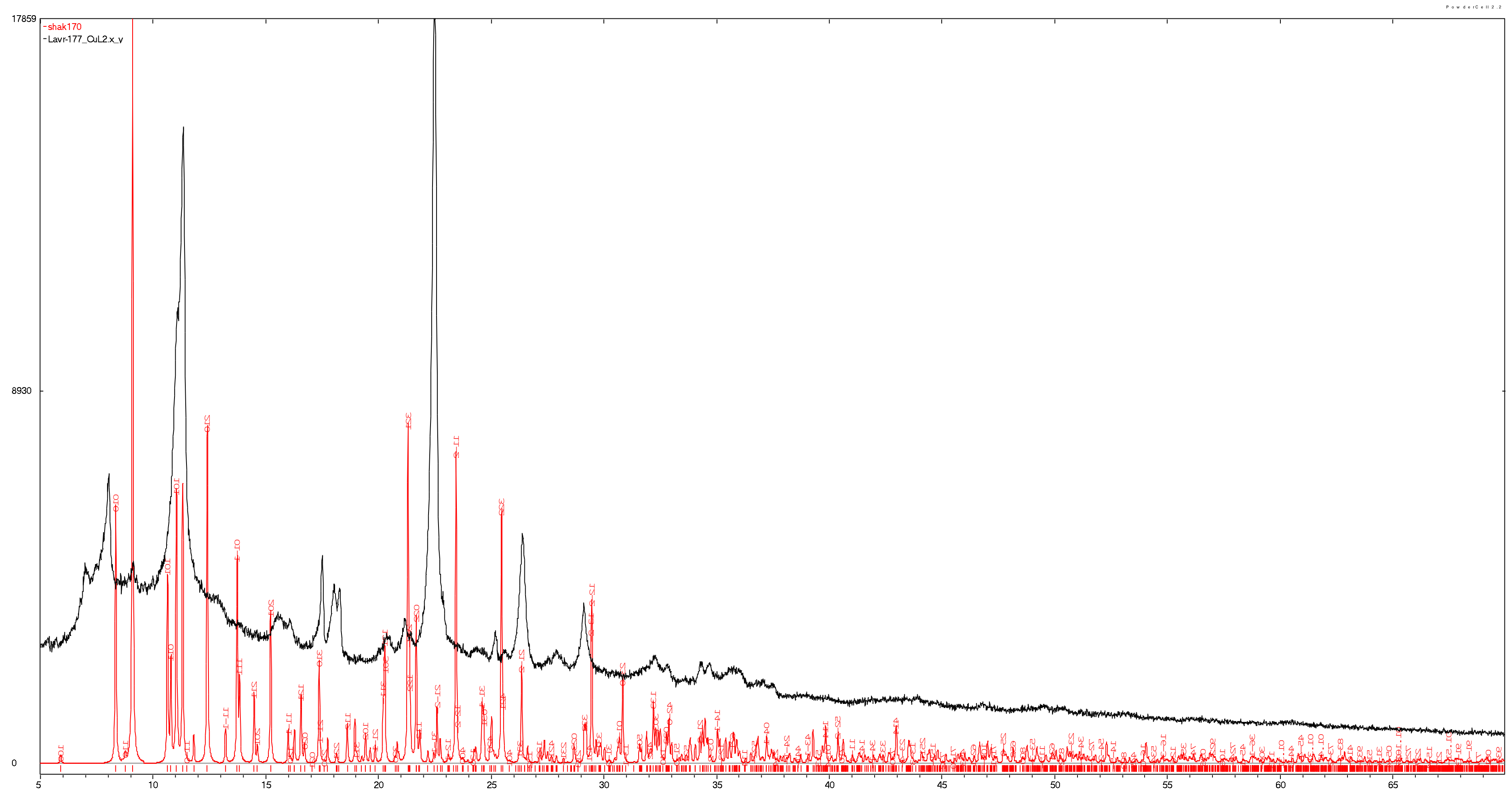

FigureS1 – Diffractograms of complexes **1** (black line) and **2** (red line).

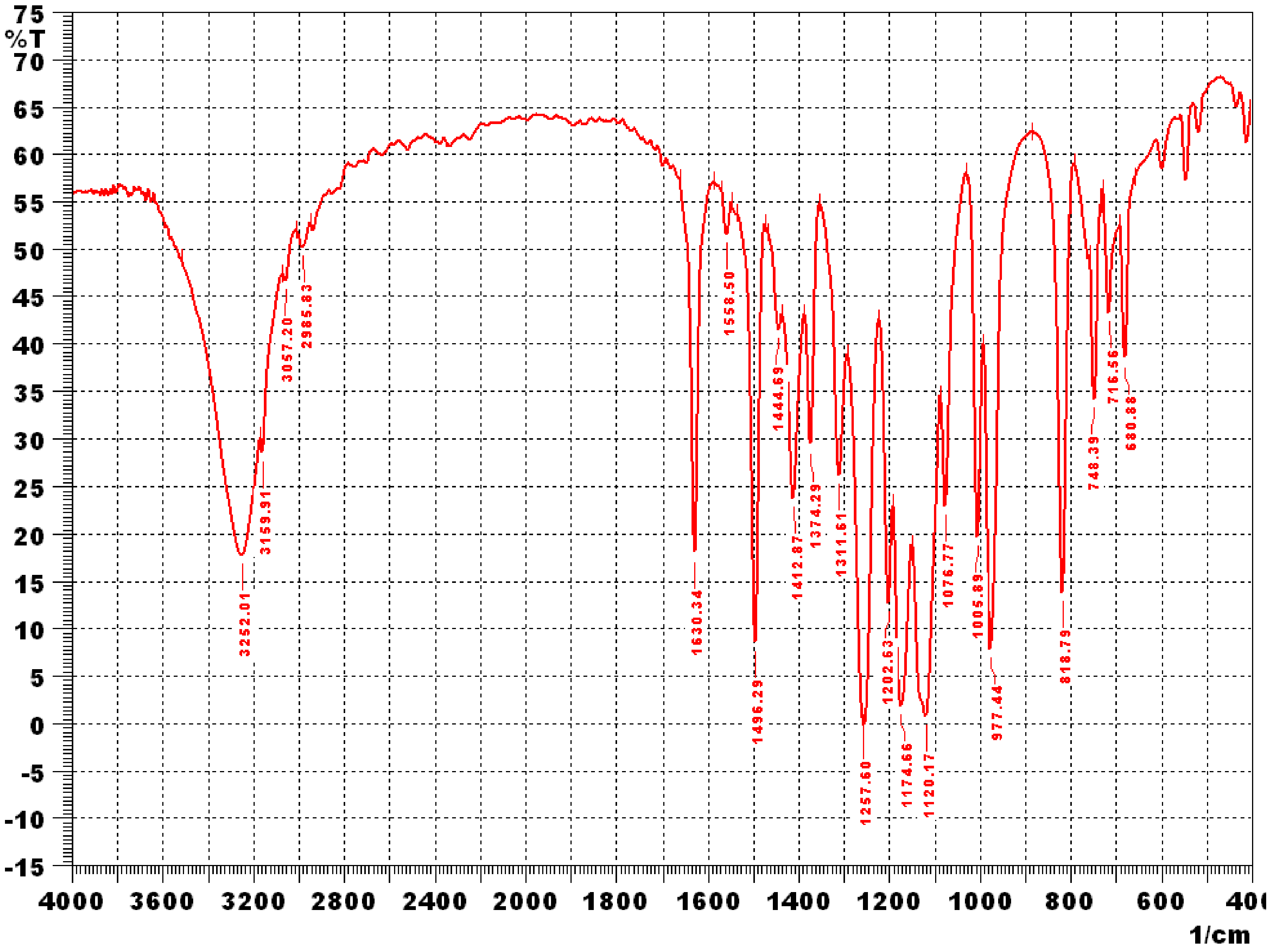

FigureS2 – IR SpectrumH_2L

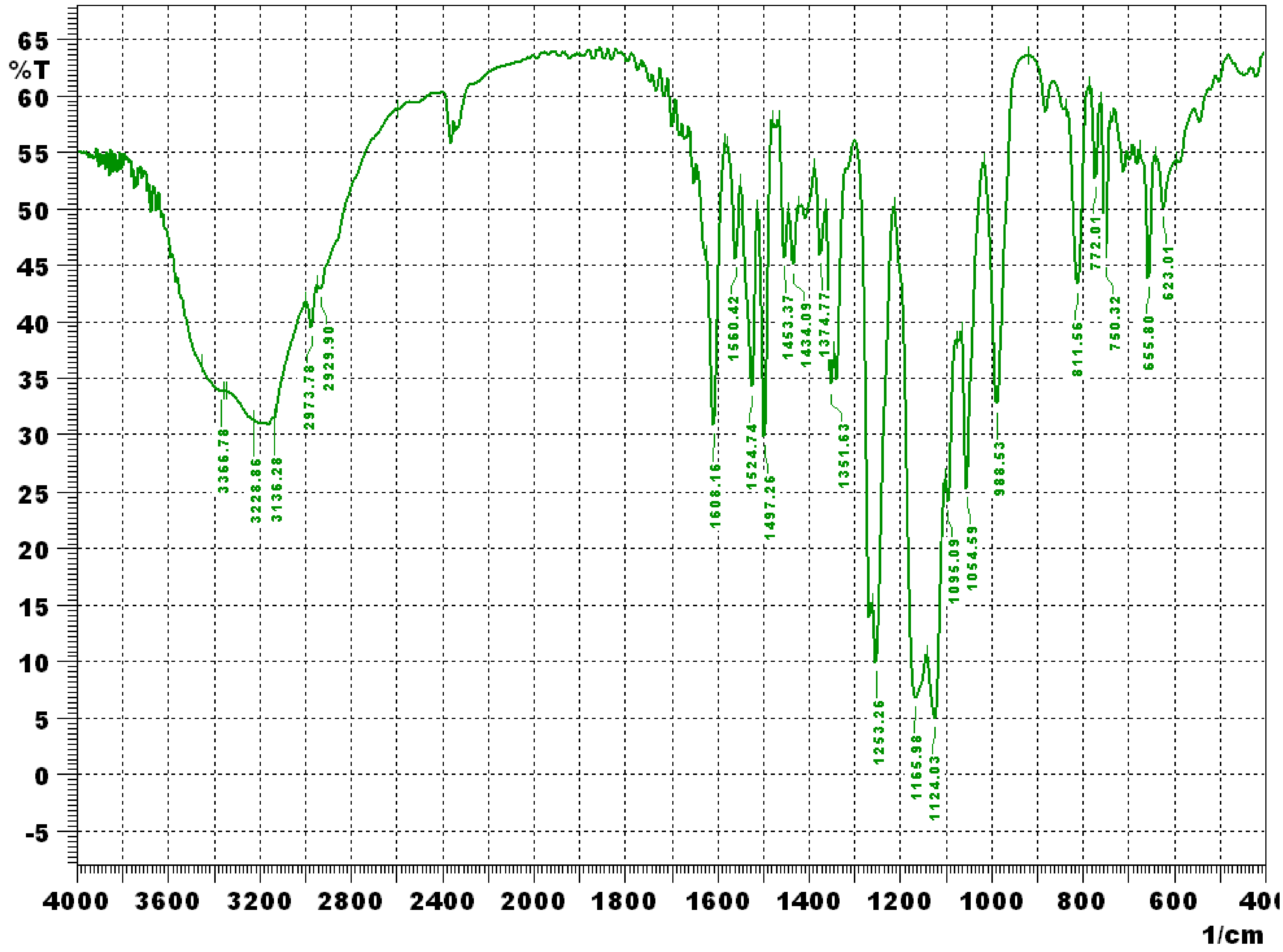

FigureS3 – IRSpectrum[Cu_2L_2]·C_2H_5OH

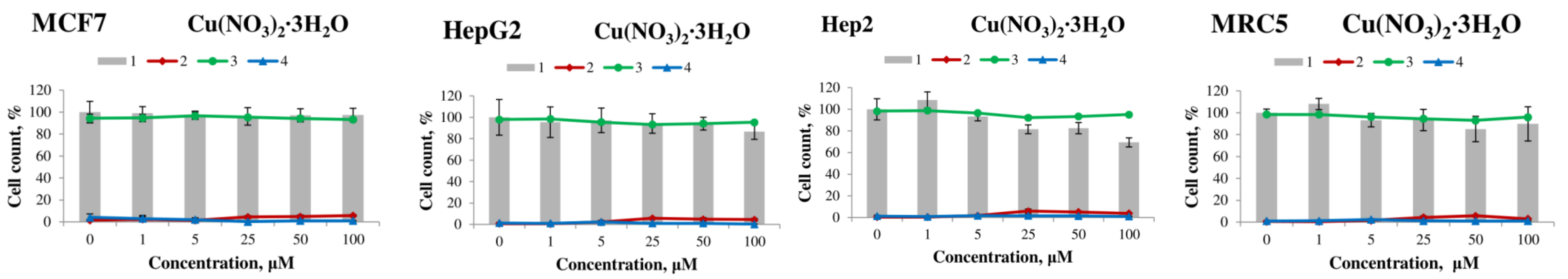

FigureS4 – Effect of $Cu(NO_3)_2 \cdot 3H_2O$ on the viability of MCF7, Hep2, HepG2 human tumor cells and MRC5 human non-tumor fibroblasts after 48 h exposure. 1 - total number of cells, 2 - dead cells, 3 - live cells, 4 - apoptotic cells
